# Changes and prognostic values of tumor-infiltrating lymphocyte subsets after primary systemic therapy in breast cancer

**DOI:** 10.1371/journal.pone.0233037

**Published:** 2020-05-13

**Authors:** Soomin Ahn, Yul Ri Chung, An Na Seo, Milim Kim, Ji Won Woo, So Yeon Park

**Affiliations:** 1 Department of Pathology, Seoul National University Bundang Hospital, Seongnam, Gyeonggi, Republic of Korea; 2 Department of Pathology, Seoul National University College of Medicine, Seoul, Republic of Korea; 3 Department of Pathology, School of Medicine, Kyungpook National University, Kyungpook National University Chilgok Hospital, Daegu, Republic of Korea; Gangnam Severance Hospital, Yonsei University College of Medicine, REPUBLIC OF KOREA

## Abstract

Tumor-infiltrating lymphocyte (TIL) levels have prognostic and predictive values in treatment-naïve breast cancers. However, there have been controversies regarding TIL subset changes and their clinical implications in post-treatment breast cancers. This study aimed to explore change and prognostic significance of TIL subset infiltration after primary systemic therapy (PST) in breast cancer. One-hundred-fifty-five patients who had residual disease after anthracycline- or anthracycline plus taxane-based PST were included. The quantities of intratumoral and stromal TIL subsets (CD8+, CD4+, and FOXP3+ TILs) in pre- and post-PST breast cancer samples, as well as changes between them, were analyzed along with their correlations with clinicopathologic features and outcome of patients. As a whole, intratumoral CD8+ and CD4+ TILs increased after PST while stromal TILs decreased. Both intratumoral and stromal FOXP3+ TILs decreased after PST. The chemo-sensitive group [residual cancer burden (RCB) class I and II] showed the same pattern of change in intratumoral CD8+ TILs as the whole group, whereas the chemo-resistant group (RCB class III) showed no significant change in intratumoral CD8+ TIL infiltration after PST. Survival analyses for each TIL subset as well as their ratios revealed that high levels of intratumoral, stromal, and total CD8+ TIL infiltration after PST were independent predictors of longer patient survival. In subgroup analyses, CD8+ TIL infiltration after PST revealed prognostic significance in the chemo-resistant group but not in the chemo-sensitive group. In conclusion, infiltration of CD8+, CD4+, and FOXP3+ TIL changed after PST in the intratumoral and stromal compartments. Especially, increase of intratumoral CD8+ TILs was associated with chemo-responsiveness. Moreover, CD8+ TIL status in residual tumors after PST may be used as a potential prognostic marker in breast cancer patients who receive PST and provide additional prognostic information to chemo-resistant group.

## Introduction

Studies of the dynamic interaction between tumor cells and the immune system have established a new era in cancer research and treatment. High levels of tumor-infiltrating lymphocytes (TILs) generally indicate a more robust anti-tumor immune response, and have been shown to be associated with favorable clinical outcomes in patients with breast cancer, especially in those with triple-negative breast cancer [1–3]. Among TIL subsets, cytotoxic CD8+ T cell infiltration is considered a key indicator of effective antitumor immunity, and high CD8+ TILs have been associated with favorable clinical outcome [4, 5]. CD4+ T cells also play critical roles in regulating many aspects of adaptive immunity, and various subsets of CD4+ T cells exhibit both pro-tumor and anti-tumor activities [6, 7]. Especially, FOXP3+ regulatory T cells, which are also known as CD4+CD25+ Tregs, have suppressive effects on the anti-tumor immunity, and have been associated with poor clinical outcome [8–10].

Primary systemic therapy (PST) is considered the standard treatment for locally advanced breast cancer, and its use has substantially increased [11]. TILs are of predictive and prognostic values in breast cancer patients treated with PST; the presence of high TILs in pretreatment biopsy samples is a significant predictor of the response to PST [12, 13] and the presence of high TILs in residual disease is an indicator of better prognosis, especially in triple-negative breast cancer (TNBC) patients [14–16]. As for TIL subsets, studies have shown that high CD8+ TIL infiltration is a good predictor of pathologic complete response (pCR) [13, 17]. Higher levels of CD4+ and FOXP3+ TILs have also been reported to predict pCR [17–19].

Comparing pre-treatment biopsies and post-PST resection samples allows for the assessment of sensitivity to chemotherapy and provides important information regarding the dynamics of TILs during therapy [20]. However, there have been conflicting results about TIL change in post-treatment breast cancers. A previous study reported a trend for higher TIL counts in post-PST breast cancer samples compared to pre-PST samples [15]. On the contrary, subsequent studies revealed decrease in TIL count in breast cancer after chemotherapy completion [21, 22]. As for TIL subsets, a limited number of studies have compared TIL subsets in breast cancer pre- and post-PST [19, 23–27], but conflicting data exist regarding the changes in each TIL subsets after PST. Moreover, PST-induced changes in TIL subsets in different histologic locations (intratumoral or stromal) have not been studied, although it has been recommended that intratumoral and stromal TILs be evaluated separately because the TIL densities in these two compartments may differ in tumors [28]. Furthermore, while the prognostic significance of TIL subset infiltration is becoming better understood in treatment-naïve breast cancer, it remains unclear in post-treatment breast cancer.

To that end, we performed this study to evaluate the changes in CD8+, CD4+, and FOXP3+ TILs in each compartment after PST, and investigated the association between TIL subset infiltration and survival in patients with breast cancer who were treated with anthracycline- or anthracycline plus taxane-based PST using pre- and post-PST specimens.

## Materials and methods

### Ethics statement

The study was approved by the institutional review board of Seoul National University Bundang Hospital (Protocol # B-1601/332-304). The requirements for informed consent from participants were waived by the institutional review board. All the specimens used in this study were obtained from archival material in the Department of Pathology. The patients’ medical records were accessed during the data collection, but all the data and tissue samples were fully anonymized before we analyzed them.

### Study population

This study included 155 patients with clinical stage II or III breast cancer who received breast conserving surgery or mastectomy after anthracycline- or anthracycline plus taxane-based PST at Seoul National University Bundang Hospital between 2004 and 2012. Patients who achieved pathologic complete response after PST and those whose pre-PST biopsies were performed at outside hospitals and were not available for study were excluded from the study. All patients were diagnosed with invasive carcinoma via core needle biopsy. Table 1 lists the patients’ clinicopathological characteristics. Of all the 155 patients, 91 (58.7%) and 64 (41.3%) had stage II and stage III disease, respectively. Seventy-three (47.1%) patients were treated with the ‘AC’ regimen (60 mg/m^2^ doxorubicin intravenously on day 1 and 600 mg/m^2^ cyclophosphamide intravenously once every three weeks for 4–6 cycles), 46 (30.5%) were treated with the ‘AC-D’ regimen (four cycles of AC followed by four cycles of 75 mg/m^2^ docetaxel), and the remaining 36 (23.2%) received the ‘AD’ regimen (50 mg/m^2^ doxorubicin intravenously on day 1 and 75 mg/m^2^ docetaxel intravenously once every three weeks for 3–6 cycles). Patients underwent definitive surgical resection 3–4 weeks after the final chemotherapy cycle. None of the patient received neoadjuvnat trastuzumab, and 30 (93.8%) of 32 patients with human epidermal growth factor receptor 2 (HER2)-positive breast cancer were treated by adjuvant trastuzuamb postoperatively. One hundred thirty-two patients (85.2%) received adjuvant radiotherapy and 118 (76.1%) received adjuvant endocrine therapy.

**Table 1:** Baseline characteristics of patients in pre-primary systemic therapy status.

Characteristics	Number (%)
Age, years	
Median (range)	46 (30–72)
Clinical stage	
II	91 (58.7)
III	64 (41.3)
Clinical T stage	
T1-T2	97 (62.6)
T3-T4	58 (37.4)
Clinical N stage	
N0	25 (16.1)
N1-N3	130 (83.9)
Histologic subtype	
IDC	137 (88.4)
ILC	5 (3.2)
Metaplastic carcinoma	4 (2.6)
Mucinous carcinoma	4 (2.6)
Others	5 (3.2)
Histologic grade	
Low	19 (12.3)
Intermediate	90 (58.1)
High	46 (29.7)
Estrogen receptor	
Negative	36 (23.2)
Positive	119 (76.8)
Progesterone receptor	
Negative	57 (36.8)
Positive	98 (63.2)
HER2 status	
Negative	123 (79.4)
Positive	32 (20.6)
Subtype	
Luminal A	56 (36.1)
Luminal B	65 (41.9)
HER2+	11 (7.1)
Triple-negative	23 (14.8)
Ki-67 proliferation index	
<20%	81 (52.3)
≥20%	74 (47.7)
P53 overexpression	
Absent	102 (65.8)
Present	53 (34.2)
PST regimen	
AC	73 (47.1)
AC-D	46 (29.7)
AD	36 (23.2)

IDC, invasive ductal carcinoma; ILC, invasive lobular carcinoma; PST, primary systemic therapy; AC, doxorubicin plus cyclophosphamide; AC-D, AC followed by docetaxel; AD, doxorubicin plus docetaxel

A pair of formalin-fixed and paraffin-embedded samples comprising pre-PST biopsy and post-PST resection samples was acquired for each patient. Clinicopathologic data were retrieved from electronic medical records. Hematoxylin and eosin-stained sections and immunohistochemically stained slides for standard biomarkers of initial biopsy and post-PST resection samples were reviewed and the following parameters recorded: clinical T stage, clinical N stage, size of tumor, histologic subtype (by World Health Organization classification), histologic grade (using the Bloom and Richardson grading system), lymphovascular invasion, estrogen receptor (ER), progesterone receptor (PR) and HER2 status, Ki-67 proliferation index, p53 overexpression, chemotherapeutic regimen, numbers of PST cycles, and pathologic T and N stages after PST. The pathologic response to PST was evaluated using the residual cancer burden (RCB) system [29]. Of the 155 post-PST samples, 7 (4.5%) cases belonged to RCB class I, 68 (43.9%) to class II, 80 (51.6%) to class III. RCB classes I and II were regarded as chemo-sensitive, responder group, and RCB class III was regarded as chemo-resistant, non-responder group.

### Immunohistochemical staining

Immunohistochemical staining was performed on pre-PST biopsy and post-PST resection samples using a BenchMark XT autostainer (Ventana Medical Systems, Tucson, AZ, USA) with an UltraView detection kit (Ventana Medical Systems). Commercially-available antibodies were used for immunohistochemical staining of TIL subsets: CD8 (clone C8/144B; ready to use; Dako, Carpinteria, CA, USA), CD4 (clone SP35; ready to use; Dako), and FOXP3 (clone 236A/E7; 1:100; Abcam, Cambridge, UK). Information regarding standard biomarkers in both pre- and post-PST specimens, including ER, PR, HER2, Ki-67, and p53, was available for most patients. For patients in whom these data were unavailable, we used the following antibodies for immunohistochemistry: ER (clone SP1; 1:100; LabVision, Fremont, CA, USA), PR (clone PgR 636; 1:70; Dako), HER2 (clone 4B5; ready to use; Ventana), p53 (clone D07; 1:600; Dako), and Ki-67 (clone MIB-1; 1:250; Dako). For ER and PR, 1% or greater staining was considered positive. For HER2, a score of 3+ on immunohistochemistry and/or the presence of gene amplification on fluorescence/silver in situ hybridization were considered positive. Breast cancer subtypes were categorized according to the 2011 St. Gallen Expert Consensus [30] based on biomarker data from the pre-treatment biopsy specimen as follows: luminal A subtype (ER+ and/or PR+, HER2-, Ki-67 <14%), luminal B subtype (ER+ and/or PR+, HER2-, Ki-67 ≥14%; ER+ and/or PR+, HER2+), HER2+ subtype (ER-, PR-, HER2+), and triple-negative subtype (ER-, PR-, HER2-).

### Quantification of TILs

To evaluate CD8+, CD4+, and FOXP3+ TIL infiltration in breast cancer before and after PST, the number of each TIL subset was counted by two pathologists (YRC and ANS) who were blinded to the clinicopathologic features of the tumors. Three high-power fields (×40 objective) were randomly selected within tumors that had a relatively homogeneous distribution of these lymphocytes. In tumors with heterogeneous infiltration of TILs, five high-power fields were chosen randomly in areas other than those with either the highest or lowest infiltration. The selected high-power fields were photographed with an OLYMPUS DP70 digital microscope camera (Olympus Optical Co., Ltd); the images were then used to count the absolute number of TILs in both intratumoral and stromal areas using the UTHSCSA Image Tool software (version 3.0, Department of Dental Diagnostic Science at The University of Texas Health Science Center, San Antonio, TX, USA). Using the same method reported by Denkert et al. [12], lymphocytes in the tumor nests or those directly contacting the tumor cells were considered intratumoral TILs; the remainders were considered stromal TILs. Average numbers of intratumoral, stromal, and total CD8+, CD4+, and FOXP3+ TILs per high-power field were recorded. Moreover, the ratios of CD8+/CD4+, FOXP3+/CD8+, and FOXP3+/CD4+ T cells were also calculated. Medians of TIL subsets and their ratios were used as cutoff values for dichotomization into high versus low groups in each analysis and values greater than median were categorized as high.

### Statistical analysis

All statistical analyses were performed using SPSS version 25.0 for Windows (IBM Corp., Armonk, NY, USA). Wilcoxon signed rank test was used to compare TIL subset infiltration in each compartment (intratumoral, stromal, and total) between matched pre- and post-PST samples. Spearman’s rank correlation tests were used to assess the associations among infiltrations of CD8+, CD4+, and FOXP3+ TILs. The associations between TIL subset infiltration or the ratios of TIL subsets and clinicopathological features were analyzed by the chi-square test or Fisher’s exact test using total counts of each TIL subset. Kaplan-Meier curves were used for survival analysis, and differences were compared using the log-rank test. Univariate and multivariate analyses were performed using the Cox proportional hazards model with a backward stepwise selection method. Hazard ratios and 95% confidence intervals were calculated for each variable. *P*-values less than 0.05 were considered statistically significant; all *p*-values were two-sided.

## Results

### Changes in TIL subsets after PST

In pre-PST biopsy samples, CD8+, CD4+, and FOXP3+ TIL infiltration was present in 152 (98.1%), 152 (98.1%), and 132 (85.2%) cases, respectively. In post-PST resection samples, all showed CD8+ and CD4+ TIL infiltration, and 128 (82.6%) exhibited FOXP3+ TIL infiltration. Total CD8+ TIL levels increased in 76 (49.0%) of 155 patients and the ratios of post-PST CD8+ TIL to pre-PST CD8+ TIL ranged from 0.02 to 60.61. CD4+ TIL levels increased after PST in 73 (47.1%) patients with the ratios ranging from 0.01 to 75.76. FOXP3+ TIL increased in 34 patients (21.9%), and the ratios varied from 0 to 12.12. Detailed data were presented in S1 Table.

**Fig 1:**
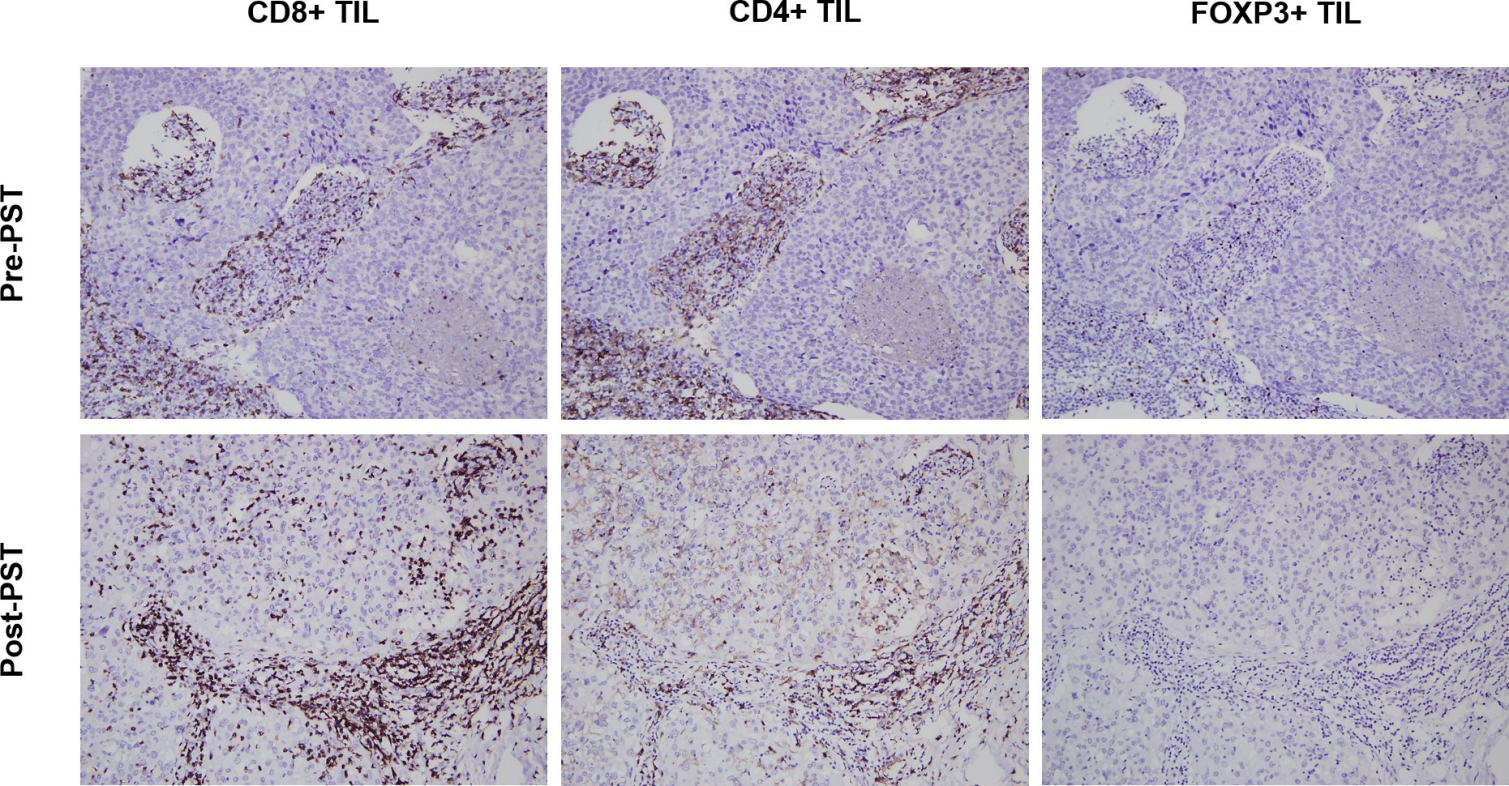
A representative chemo-responsive case showing changes in the infiltration of CD8+, CD4+, and FOXP3+ tumor-infiltrating lymphocytes after primary systemic therapy. CD8+ tumor-infiltrating lymphocytes (TILs) increased significantly after primary systemic therapy (PST), especially in the intratumoral compartment. CD4+ TILs increased slightly in the intratumoral compartment, but decreased in the stromal compartment after PST. FOXP3+ TILs decreased significantly with no identifiable cells in the figure (Original magnification, x200).

Paired analyses of TIL subsets before and after PST are shown in Table 2. While the total number of CD8+ and CD4+ TILs did not show a statistically significant change in their numbers after PST (*p* = 0.546, *p* = 0.239, respectively), FOXP3+ TILs decreased significantly after PST (*p*<0.001) (Fig 1). As for individual compartments, the quantities of intratumoral CD8+ and CD4+ TILs increased after PST (*p* = 0.013, *p* = 0.013, respectively) while stromal TILs decreased (*p* = 0.015, *p* = 0.005, respectively). FOXP3+ TILs showed a different pattern, with decreased intratumoral and stromal TILs after PST (all *p*<0.001).

**Table 2:** Paired analyses of tumor-infiltrating lymphocytes before and after primary systemic therapy.

Group	TIL	Pre-PST	Post-PST	p-value
Whole group (n = 155)	CD8+ TIL, intratumoral	7.7 (2.0–28.0)	13.7 (3.3–34.0)	0.013
	CD8+ TIL, stromal	35.0 (15.7–94.0)	27.3 (11.7–53.3)	0.015
	CD8+ TIL, total	45.5 (19.0–129.3)	39.7 (19.3–87.3)	0.546
	CD4+ TIL, intratumoral	28.7 (3.0–90.3)	42.0 (1.3–128.0)	0.013
	CD4+ TIL, stromal	87.3 (21.7–250.7)	76.0 (30.3–160.0)	0.005
	CD4+ TIL, total	114.7 (28.3–344.3)	120.0 (37.6–317.7)	0.239
	FOXP3+ TIL, intratumoral	3.3 (0.3–9.7)	1.0 (0.0–3.7)	<0.001
	FOXP3+ TIL, stromal	11.0 (2.3–26.0)	2.0 (0.5–5.3)	<0.001
	FOXP3+ TIL, total	14.7 (2.3–35.7)	3.3 (1.0–7.3)	<0.001
Chemo-sensitive group* (n = 75)	CD8+ TIL, intratumoral	8.0 (2.0–32.3)	16.3 (3.0–57.0)	0.008
	CD8+ TIL, stromal	40.3 (14.0–92.0)	31.7 (11.7–94.7)	0.980
	CD8+ TIL, total	49.3 (16.0–126.3)	48.7 (20.0–157.0)	0.252
	CD4+ TIL, intratumoral	33.0 (2.0–104.0)	49.0 (1.0–161.3)	0.055
	CD4+ TIL, stromal	106.3 (18.0–313.7)	86.7 (34.0–174.3)	0.171
	CD4+ TIL, total	139.7 (25.3–402.7)	129.7 (37.7–346.0)	0.788
	FOXP3+ TIL, intratumoral	3.7 (0.3–16.0)	1.3 (0.0–7.0)	0.001
	FOXP3+ TIL, stromal	11.7 (4.0–34.3)	2.7 (1.0–9.3)	<0.001
	FOXP3+ TIL, total	16.7 (4.7–47.3)	4.7 (2.0–13.7)	<0.001
Chemo-resistant group* (n = 80)	CD8+ TIL, intratumoral	7.2 (2.1–26.9)	12.8 (3.4–25.3)	0.370
	CD8+ TIL, stromal	30.3 (19-0-100.3)	24.7 (10.5–42.7)	<0.001
	CD8+ TIL, total	37.5 (21.0–134.2)	37.7 (18.1–63.4)	0.034
	CD4+ TIL, intratumoral	27.0 (3.1–78.8)	39.7 (2.2–108.7)	0.123
	CD4+ TIL, stromal	86.3 (30.2–247.9)	65.7 (25.4–136.8)	0.010
	CD4+ TIL, total	110.8 (36.3–323.0)	106.2 (40.9–255.8)	0.153
	FOXP3+ TIL, intratumoral	3.3 (0.3–7.7)	1.0 (0.0–2.3)	<0.001
	FOXP3+ TIL, stromal	11.0 (1.2–19.3)	1.3 (0.1–3.3)	<0.001
	FOXP3+ TIL, total	14.2 (1.7–28.7)	2.7 (0.4–5.3)	<0.001

*P* values are calculated by Wilcoxon signed rank test. Data represent cell count per high power filed and were presented as median (interquartile range).

*Chemo-sensitive group indicates residual cancer burden (RCB) classes I & II, and chemo-resistant group indicates RCB class III.

TIL, tumor-infiltrating lymphocyte; PST, primary systemic therapy

### Change of TIL subsets after PST according to chemo-responsiveness

Paired analyses in subgroups classified by chemo-responsiveness (Table 2) revealed that the chemo-sensitive group showed the same pattern of change in intratumoral CD8+ TILs as in the entire group (*p* = 0.008) albeit with borderline statistical significance for intratumoral CD4+ TILs (*p* = 0.055). However, stromal CD8+ and CD4+ TIL infiltration did not change significantly after PST in this group (*p* = 0.980, *p* = 0.171, respectively). In the chemo-resistant group, stromal CD8+ and CD4+ TILs followed the same pattern as the whole group (*p*<0.001, *p* = 0.010, respectively), whereas intratumoral CD8+ and CD4+ TILs did not show a statistically significant change in their numbers after PST (*p* = 0.370, *p* = 0.123, respectively). FOXP3+ TILs (intratumoral, stromal, and total) showed a similar change pattern irrespective of chemo-responsiveness (all *p<* 0.005).

### Relationship between TIL subset infiltration after PST and clinicopathological characteristics of tumor

Next, we evaluated the relationship between the infiltration of TIL subsets in total and clinicopathologic features of tumors after PST. High CD8+ TIL infiltration after PST was more frequently found in ER- and PR-negative cancers (*p =* 0.031 and *p =* 0.019, respectively) and in tumors with p53 overexpression (*p =* 0.012). High CD4+ TIL infiltration after PST was associated with ER negativity (*p* = 0.031), high Ki-67 index (*p =* 0.004) and p53 overexpression (*p* = 0.031), while high FOXP3+ infiltration after PST was associated with negative node status after PST (*p =* 0.004), low RCB class (*p* = 0.046), ER negativity (*p =* 0.005), high Ki-67 index (*p*<0.001), and p53 overexpression (*p =* 0.034) (Table 3). Infiltrations of CD8+, CD4+, and FOXP3+ TILs showed moderate correlation with one another (CD8+ vs. CD4+ TIL, rho = 0.627, *p*<0.001; CD4+ vs. FOXP3+ TIL, rho = 0.623, *p*<0.001; CD8+ vs. FOXP3+ TIL, rho = 0.498, *p*<0.001). Thus, we also analyzed the relationship between the ratios of T cell subsets (CD8+/CD4+, FOXP3+/CD8+, and FOXP+/CD4+ T cell) and clinicopathologic features after treatment (S2 Table). In post-PST resection specimens, a high FOXP3+/CD8+ T cell ratio was associated with an absence of nodal metastasis after PST (*p =* 0.011) and a high Ki-67 index (*p =* 0.011). A high FOXP3+/CD4+ TIL ratio was related to a low ypT stage (*p =* 0.013), negative node status after PST (*p =* 0.004), low RCB class (*p* = 0.002), ER negativity (*p =* 0.001), and p53 overexpression (*p =* 0.012).

**Table 3:** Relationship between infiltration of tumor-infiltrating lymphocyte subsets after primary systemic therapy and post-treatment clinicopathological features of tumor.

Clinicopathologic characteristics	Post-PST CD8+ TILs		p-value	Post-PST CD4+ TILs		p-value	Post-PST FOXP3+ TILs		p-value
	Low	High		Low	High		Low	High	
	No. (%)	No. (%)		No. (%)	No. (%)		No. (%)	No. (%)	
ypT stage			0.573			0.376			0.089
T1	38 (48.7)	41 (53.2)		37 (47.4)	42 (54.5)		36 (44.4)	43 (58.1)	
T2-T4	40 (51.3)	36 (46.8)		41 (52.6)	35 (45.5)		45 (55.6)	31 (41.9)	
ypN stage			0.309			0.263			0.004
N0	25 (32.1)	19 (24.7)		19 (24.4)	25 (32.5)		15 (18.5)	29 (39.2)	
N1-N3	53 (67.9)	58 (75.3)		59 (75.6)	52 (67.5)		66 (81.5)	45 (60.8)	
RCB class			0.576			0.378			0.046
I-II	36 (46.2)	39 (50.6)		35 (44.9)	40 (51.9)		33 (40.7)	42 (56.8)	
III	42 (53.8)	38 (49.4)		43 (55.1)	37 (48.1)		48 (59.3)	32 (43.2)	
Estrogen receptor			0.031			0.031			0.005
Negative	12 (15.4)	23 (29.9)		12 (15.4)	23 (29.9)		11 (13.6)	24 (32.4)	
Positive	66 (84.6)	54 (70.1)		66 (84.6)	55 (70.1)		70 (86.4)	50 (67.6)	
Progesterone receptor			0.019			0.172			0.142
Negative	27 (34.6)	41 (53.2)		30 (38.5)	38 (49.4)		31 (38.3)	37 (50.0)	
Positive	51 (65.4)	36 (46.8)		48 (61.5)	39 (50.6)		50 (61.7)	37 (50.0)	
HER2 status			0.967			0.452			0.494
Negative	62 (79.5)	61 (79.2)		60 (76.9)	63 (81.8)		66 (81.5)	57 (77.0)	
Positive	16 (20.5)	16 (20.8)		18 (23.1)	15 (18.2)		15 (18.5)	17 (23.0)	
Ki-67 index			0.140			0.004			<0.001
Low (<20%)	60 (76.9)	51 (66.2)		64 (82.1)	47 (61.0)		71 (87.7)	40 (54.1)	
High (≥20%)	18 (23.1)	26 (33.8)		14 (17.9)	30 (39.0)		10 (12.3)	34 (45.9)	
P53 overexpression			0.012			0.031			0.034
Absent	62 (79.5)	47 (61.0)		61 (78.2)	48 (62.3)		63 (77.8)	46 (62.2)	
Present	16 (20.5)	30 (39.0)		17 (21.8)	29 (37.7)		18 (22.2)	28 (37.8)	

*P* values were calculated by the chi-square or Fisher’s exact test.

PST, primary systemic therapy; TIL, tumor-infiltrating lymphocyte; HER2, human epidermal growth factor receptor 2

### Prognostic significance of TIL subsets

**Fig 2:**
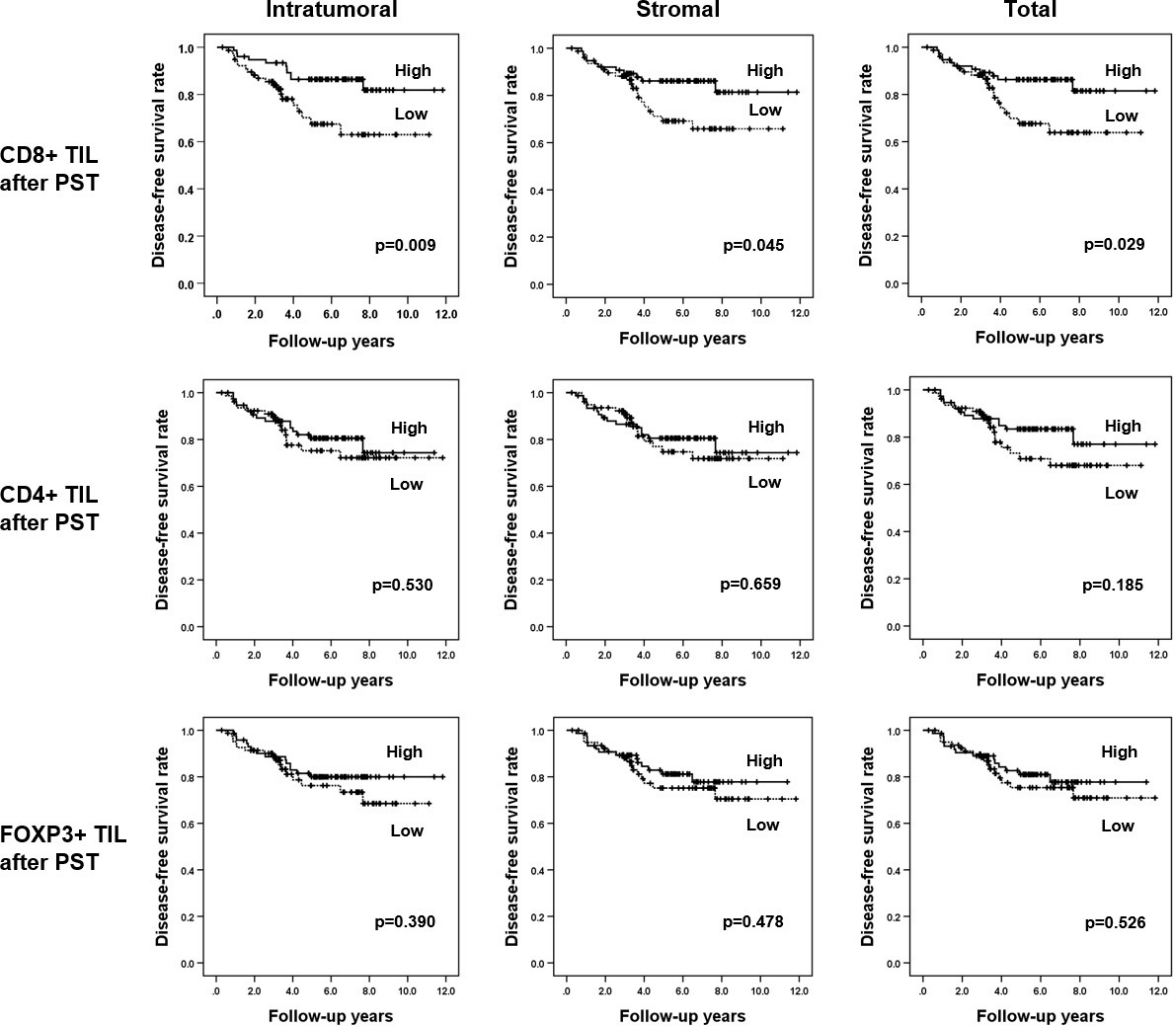
Kaplan-Meier survival analyses according to the level of tumor-infiltrating lymphocyte subsets in each compartment in post-primary systemic therapy samples. High levels of the intratumoral, stromal, and total CD8+ tumor-infiltrating lymphocyte (TIL) infiltration after primary systemic therapy (PST) are associated with longer patient disease-free survival, whereas levels of CD4+ and FOXP3+ TIL filtration after PST have no prognostic significance.

Most patients received standard treatment and regular follow-up; the mean follow-up period was 62 months (range, 3–142 months). Kaplan-Meier survival curves for all the TIL subset variables using total count (pre-PST CD8+, CD4+, and FOXP3+ TILs; post-PST CD8+, CD4+, and FOXP3+ TILs; pre-PST FOXP3+/CD8+, FOXP3+/CD4+, and CD8+/CD4+ TIL ratios; post-PST FOXP3+/CD8+, FOXP3+/CD4+, and CD8+/CD4+ TIL ratios; and post-PST CD8+/pre-PST CD8+, post-PST CD4+/pre-PST CD4+, post-PST FOXP3+/pre-PST FOXP3 TIL ratios) revealed that only high CD8+ TIL infiltration after PST was significantly associated with a longer disease-free survival (*p =* 0.029, log-rank test; Fig 2; S3 Table). High CD8+/CD4+ TIL ratio after PST tended to be associated with increased disease-free survival of the patients (*p* = 0.072). When analyzing in each compartment, high intratumoral and stromal CD8+ TIL infiltrations after PST were associated with longer disease-free survival (*p =* 0.009 and *p* = 0.045, log-rank test, respectively; Fig 2). However, post-PST CD4+ and FOXP3+ TIL infiltration in each compartment had no prognostic significance (Fig 2).

Table 4 shows the results of univariate and multivariate analyses of disease-free survival using the Cox proportional hazards model in the entire patient group. On univariate analysis in the whole group, a high ypT stage, high RCB class and high Ki-67 proliferation index were found to be poor prognostic factors (*p =* 0.039, *p* = 0.030, and *p =* 0.017, respectively) while positive ER status and high post-PST CD8+ TILs were good prognostic indicators (*p =* 0.007 and *p =* 0.033, respectively). On multivariate analysis, RCB class, ER status and post-PST CD8+ TILs remained independent prognostic factors (*p =* 0.002, *p*<0.001, and *p =* 0.017, respectively).

**Table 4:** Univariate and multivariate analyses of disease-free survival.

Variable	Category	Univariate analysis			Multivariate analysis		
		HR	95% CI	p-value	HR	95% CI	p-value
ypT stage	T1 vs. T2-4	2.176	1.041–4.546	0.039	1.837	0.706–4.779	0.213
ypN stage	N0 vs. N1-3	1.914	0.734–4.989	0.184	1.977	0.679–5.756	0.211
RCB class	I-II vs. III	2.365	1.088–5.140	0.030	3.883	1.674–9.003	0.002
Post-PST ER status	Negative vs. Positive	0.369	0.179–0.762	0.007	0.187	0.085–0.414	<0.001
Post-PST PR status	Negative vs. Positive	0.500	0.245–1.020	0.057	0.595	0.222–1.594	0.302
Post-PST HER2 status	Negative vs. Positive	0.971	0.398–2.369	0.949	-	-	-
Post-PST Ki-67 index	<20% vs. ≥20%	2.372	1.169–4.813	0.017	1.607	0.587–4.401	0.356
Post-PST CD8+ TIL (total)	Low vs. High	0.447	0.213–0.938	0.033	0.402	0.190–0.851	0.017
Post-PST CD4+ TIL (total)	Low vs. High	0.618	0.301–1.268	0.190	-	-	-
Post-PST FOXP3+ TIL (total)	Low vs. High	0.795	0.391–1.618	0.527	-	-	-

HR, hazard ratio; CI, confidence interval; RCB, residual cancer burden; ER, estrogen receptor; PR, progesterone receptor; HER2, human epidermal growth factor receptor 2; PST, primary systemic therapy; TIL, tumor-infiltrating lymphocyte

Subgroup analysis by hormone receptor status revealed that high CD8+ TILs in residual tumors were of prognostic significance in the hormone receptor-positive subgroup as well as in the hormone receptor-negative subgroup (*p* = 0.037 and *p =* 0.019, respectively, log-rank test). On subgroup analyses according to breast cancer subtype, post-PST CD8+ TIL infiltration was associated with patients’ survival in HER2+ subtype (*p* = 0.174 for luminal A; *p* = 0.192 for luminal B; *p* = 0.033 for HER2+; *p* = 0.154 for triple-negative subtype, log-rank test).

**Fig 3:**
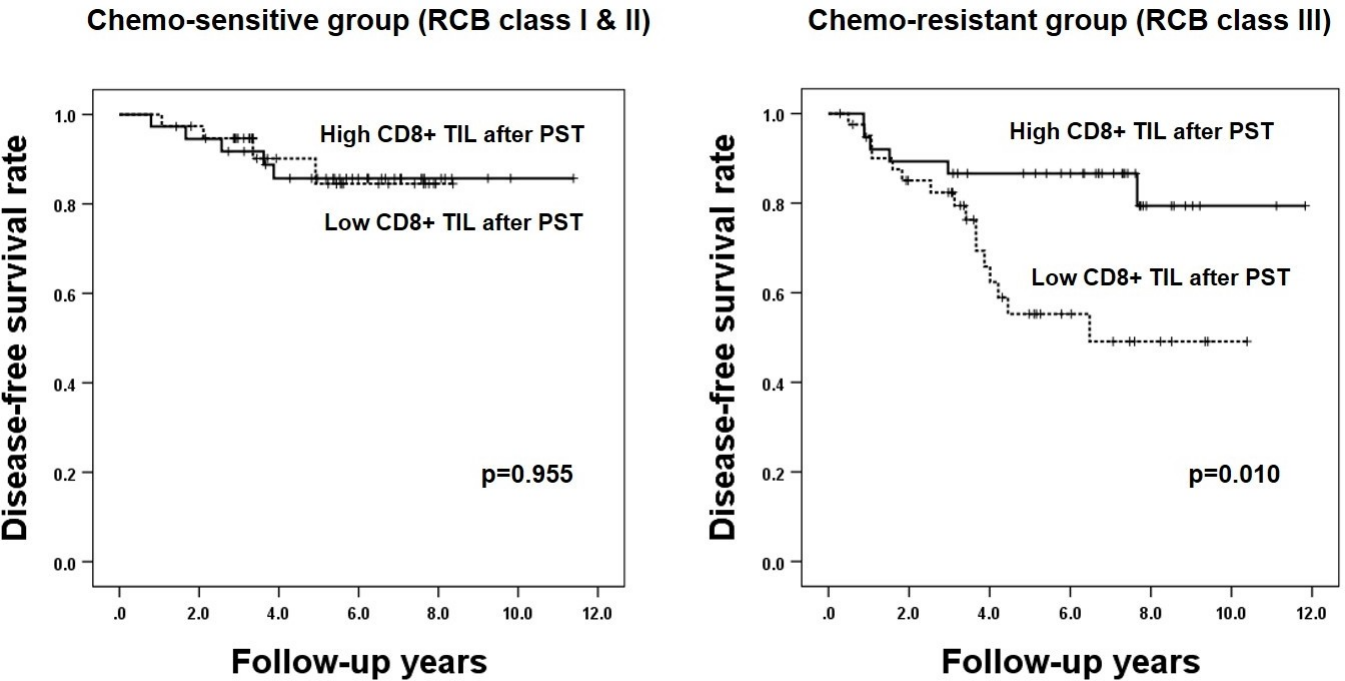
Kaplan-Meier survival analyses based on level of CD8+ tumor-infiltrating lymphocyte infiltration after primary systemic therapy according to chemo-responsiveness. High level of the CD8+ tumor-infiltrating lymphocyte (TIL) infiltration after primary systemic therapy (PST) is associated with longer patient disease-free survival in chemo-resistant group (RCB class III), but not in the chemo-sensitive group (RCB class I & II).

Finally, subgroup analysis according to chemo-responsiveness based on RCB class, CD8+ TIL infiltration after PST was associated with survival of the patients in the chemo-resistant subgroup, but not in the chemo-sensitive subgroup (RCB class I & II, *p* = 0.955; RCB class III, *p* = 0.010, log-rank test; Fig 3).

## Discussion

We explored changes in TIL subsets including CD8+, CD4+, and FOXP3+ T cells after PST, and found that total CD8+ and CD4+ TIL infiltration did not show significant change after PST, while FOXP3+ TILs decreased. So far, several studies have investigated PST-induced changes in TIL subsets in breast cancer [19, 23–27]. Ladoire et al. observed a higher infiltration of CD8+ TILs and lower infiltration of FOXP3+ TILs after PST, but they used grading system for evaluation of TILs instead of direct counting [25]. Garcia-Martinez et al. reported that CD8+ TILs increased, CD4+ TILs decreased, and FOXP3+ TILs remained unchanged after PST, using 2 mm-sized tissue microarrays for evaluation of TILs [19]. On the other hand, Ruffel et al. observed that the proportion of CD8+ TILs was unchanged and that of CD4+ TILs decreased, which produced a higher CD8+/CD4+ TIL ratio in breast cancer treated with PST [27]. However, in their study, comparison was done with treatment-naïve breast cancers, not with matched pre-PST samples. The study by Miyashita et al. was confined to TNBC and evaluated only stromal TILs; they demonstrated that CD8+ TILs increased after PST in 69% (54/78) of patients, while FOXP3+ TILs increased in 45% [26]. Hence, investigations of TIL subset changes have produced conflicting results, which may be due–at least in part–to different methodological approaches. For this reason, direct comparison of the results of the aforementioned studies and ours is not possible. A further large-scale study in an evenly-treated population is warranted to validate the results of this study.

Besides changes in TIL subsets, we also focused on the localization (intratumoral and stromal) of each TIL subset after PST. The number of intratumoral CD8+ and CD4+ TILs increased after PST, while their stromal counterparts decreased. Notably, intratumoral CD8+ TILs increased significantly after PST in the chemo-sensitive group. In contrast, there was no significant change in intratumoral CD8+ TILs in the chemo-resistant group. Based on these findings, we postulated that CD8+ TILs move from stromal to intratumoral compartments during PST, especially in chemo-sensitive tumors. The mechanism underlying TIL subset change after PST can be partly explained by the induction of specific immune responses by chemotherapeutic agents [31]. In an experimental breast cancer mouse model, doxorubicin treatment enhanced tumor antigen-specific proliferation of CD8+ T cells in tumor-draining lymph nodes and increased the infiltration of activated, interferon-γ-producing CD8+ TILs [32]. Taxanes have also been found to have an immunomodulatory effect on immune cells, which leads to the activation of innate and adaptive immunity [33].

Our analyses on prognostic significance of TIL subset infiltration in pre- and post-PST samples of breast cancer patients revealed that high CD8+ TIL infiltration after PST is associated with a favorable clinical outcome of the patients. We also observed that CD8+ TIL subset infiltration into both the intratumoral and stromal compartments after PST carries prognostic significance. CD8+ TILs are a key component of tumor-specific cellular immunity, and their prognostic value is well documented in primary operable (PST-naïve) breast cancer [4, 5]. On the other hand, there have been a relatively small number of studies that investigated the prognostic significance of TIL subsets in breast cancer following PST. In a study by Ladoire et al., high CD8+ and low FOXP3+ TILs after PST were significantly associated with longer disease-free and overall survival [25]. In a study by Miyashita et al., high CD8+ TIL levels and a high CD8+/FOXP3+ T cell ratio in residual tumors were found as indicators of a favorable clinical outcome in TNBC patients who underwent PST [26]. In subgroup analyses, our study showed the prognostic value of CD8+ TILs after PST both in the hormone receptor-positive and hormone receptor-negative breast cancers. As for breast cancer subtype, we demonstrated prognostic significance of CD8+ TILs after PST only in the HER2+ subtype. However, the analyses have a limitation due to a small number of cases in each subtype and thus, the results should be interpreted cautiously.

In further subgroup analyses, we showed the prognostic value of CD8+ TILs after PST in chemo-resistant group (RCB III) but not in chemo-responsive group (RCB I and II). Recently, Luen et al. reported that prognostic effect of residual disease TILs assessed by hematoxylin and eosin-stained sections significantly differed by RCB class [16]. However, on the contrary to our result, they showed more positive prognostic effect in RCB class II than in RCB class III [16]. Although the results are quite different, these two studies have significance in that TIL density or CD8+ TIL count provides further prognostic information to RCB class. Comparative studies will be needed in a larger cohort of patients with breast cancer to confirm our findings.

In the present study, we observed a significant decrease in FOXP3+ TILs after PST in both intratumoral and stromal compartments, irrespective of chemo-responsiveness. Previously, Liu et al. reported a significant decrease in peritumoral FOXP3+ TILs in TNBC and HER2+ tumors post-PST, but not in hormone receptor-positive tumors; furthermore, FOXP3+ TILs within tumor bed remained stable [23]. With respect to prognosis, they observed that a high infiltration of FOXP3+ TILs within tumor bed was a predictor of poorer disease-free and overall survival [23]. In our study, FOXP3+ TILs decreased after PST and their status after PST were not found to be prognostic. The dynamics and significance of FOXP3+ TILs in breast cancer after PST remain to be further investigated.

There were several limitations in our study. First, the status of TILs in pre-PST biopsy specimens may not reflect that of the entire tumor due to a sampling issue. Furthermore, we selected only some areas to evaluate the status of TIL subset infiltration. Scanning of the whole slide of the tumor and digital analyses of TIL subsets in each compartment may be a next step to extend this study. Second, as we did not include patients who had no residual tumors after PST because our study design was to evaluate TIL subset status in intratumoral and stromal compartments of such residual tumors, direct comparisons with previous studies that included patients who achieved pCR are not feasible.

In conclusion, we found that infiltration of CD8+, CD4+, and FOXP3+ TIL changed after PST in the intratumoral and stromal compartments. Especially, intratumoral CD8+ TILs increased after PST in chemo-sensitive tumors. High CD8+ TIL infiltration after PST was significantly associated with increased disease-free survival, especially in the chemo-resistant group, suggesting that CD8+ TIL status in residual tumors after PST can serve as a useful prognostic marker in breast cancer patients who undergo PST and provide additional prognostic information to the chemo-resistant group.

## Supporting information

S1 Table Data of clinicopathologic features and tumor-infiltrating lymphocyte subsets included in the analysis. (XLSX)

S2 Table Relationship between ratios of tumor-infiltrating lymphocyte subsets after primary systemic therapy and clinicopathological characteristics. (DOCX)

S3 Table Survival analyses using TIL subset variables using total count. (DOCX)

## References

[1] Loi S, Sirtaine N, Piette F, Salgado R, Viale G, Van Eenoo F, et al Prognostic and predictive value of tumor-infiltrating lymphocytes in a phase III randomized adjuvant breast cancer trial in node-positive breast cancer comparing the addition of docetaxel to doxorubicin with doxorubicin-based chemotherapy: BIG 02–98. Journal of clinical oncology: official journal of the American Society of Clinical Oncology. 2013;31(7):860–7. 10.1200/JCO.2011.41.0902 . 23341518

[2] Loi S, Michiels S, Salgado R, Sirtaine N, Jose V, Fumagalli D, et al Tumor infiltrating lymphocytes are prognostic in triple negative breast cancer and predictive for trastuzumab benefit in early breast cancer: results from the FinHER trial. Ann Oncol. 2014;25(8):1544–50. 10.1093/annonc/mdu112 . 24608200

[3] Ibrahim EM, Al-Foheidi ME, Al-Mansour MM, Kazkaz GA. The prognostic value of tumor-infiltrating lymphocytes in triple-negative breast cancer: a meta-analysis. Breast cancer research and treatment. 2014;148(3):467–76. 10.1007/s10549-014-3185-2 . 25361613

[4] Mahmoud SM, Paish EC, Powe DG, Macmillan RD, Grainge MJ, Lee AH, et al Tumor-infiltrating CD8+ lymphocytes predict clinical outcome in breast cancer. J Clin Oncol. 2011;29(15):1949–55. 10.1200/JCO.2010.30.5037 . 21483002

[5] Ali HR, Provenzano E, Dawson SJ, Blows FM, Liu B, Shah M, et al Association between CD8+ T-cell infiltration and breast cancer survival in 12,439 patients. Ann Oncol. 2014;25(8):1536–43. 10.1093/annonc/mdu191 . 24915873

[6] Kim HJ, Cantor H. CD4 T-cell subsets and tumor immunity: the helpful and the not-so-helpful. Cancer immunology research. 2014;2(2):91–8. 10.1158/2326-6066.CIR-13-0216 . 24778273

[7] Schmidt M, Weyer-Elberich V, Hengstler JG, Heimes AS, Almstedt K, Gerhold-Ay A, et al Prognostic impact of CD4-positive T cell subsets in early breast cancer: a study based on the FinHer trial patient population. Breast cancer research: BCR. 2018;20(1):15 10.1186/s13058-018-0942-x 29482642 PMC5827982

[8] Mahmoud SM, Paish EC, Powe DG, Macmillan RD, Lee AH, Ellis IO, et al An evaluation of the clinical significance of FOXP3+ infiltrating cells in human breast cancer. Breast Cancer Res Treat. 2011;127(1):99–108. 10.1007/s10549-010-0987-8 . 20556505

[9] Bates GJ, Fox SB, Han C, Leek RD, Garcia JF, Harris AL, et al Quantification of regulatory T cells enables the identification of high-risk breast cancer patients and those at risk of late relapse. J Clin Oncol. 2006;24(34):5373–80. 10.1200/JCO.2006.05.9584 . 17135638

[10] Chung YR, Kim HJ, Jang MH, Park SY. Prognostic value of tumor infiltrating lymphocyte subsets in breast cancer depends on hormone receptor status. Breast cancer research and treatment. 2017;161(3):409–20. 10.1007/s10549-016-4072-9 . 27913931

[11] Thompson AM, Moulder-Thompson SL. Neoadjuvant treatment of breast cancer. Ann Oncol. 2012;23 Suppl 10:x231–6. 10.1093/annonc/mds324 . 22987968 PMC6278992

[12] Denkert C, Loibl S, Noske A, Roller M, Muller BM, Komor M, et al Tumor-associated lymphocytes as an independent predictor of response to neoadjuvant chemotherapy in breast cancer. Journal of clinical oncology: official journal of the American Society of Clinical Oncology. 2010;28(1):105–13. 10.1200/JCO.2009.23.7370 . 19917869

[13] Mao Y, Qu Q, Zhang Y, Liu J, Chen X, Shen K. The value of tumor infiltrating lymphocytes (TILs) for predicting response to neoadjuvant chemotherapy in breast cancer: a systematic review and meta-analysis. PLoS One. 2014;9(12):e115103 10.1371/journal.pone.0115103 25501357 PMC4264870

[14] Dieci MV, Criscitiello C, Goubar A, Viale G, Conte P, Guarneri V, et al Prognostic value of tumor-infiltrating lymphocytes on residual disease after primary chemotherapy for triple-negative breast cancer: a retrospective multicenter study. Annals of oncology: official journal of the European Society for Medical Oncology. 2014;25(3):611–8. 10.1093/annonc/mdt556 24401929 PMC3933248

[15] Pelekanou V, Carvajal-Hausdorf DE, Altan M, Wasserman B, Carvajal-Hausdorf C, Wimberly H, et al Effect of neoadjuvant chemotherapy on tumor-infiltrating lymphocytes and PD-L1 expression in breast cancer and its clinical significance. Breast cancer research: BCR. 2017;19(1):91 10.1186/s13058-017-0884-8 28784153 PMC5547502

[16] Luen SJ, Salgado R, Dieci MV, Vingiani A, Curigliano G, Gould RE, et al Prognostic implications of residual disease tumor-infiltrating lymphocytes and residual cancer burden in triple-negative breast cancer patients after neoadjuvant chemotherapy. Annals of oncology: official journal of the European Society for Medical Oncology. 2019;30(2):236–42. 10.1093/annonc/mdy547 . 30590484

[17] Seo AN, Lee HJ, Kim EJ, Kim HJ, Jang MH, Lee HE, et al Tumour-infiltrating CD8+ lymphocytes as an independent predictive factor for pathological complete response to primary systemic therapy in breast cancer. British journal of cancer. 2013;109(10):2705–13. 10.1038/bjc.2013.634 24129232 PMC3833219

[18] Lee HJ, Seo JY, Ahn JH, Ahn SH, Gong G. Tumor-associated lymphocytes predict response to neoadjuvant chemotherapy in breast cancer patients. J Breast Cancer. 2013;16(1):32–9. 10.4048/jbc.2013.16.1.32 PubMed Central PMCID: PMC3625767. 23593079 PMC3625767

[19] Garcia-Martinez E, Gil GL, Benito AC, Gonzalez-Billalabeitia E, Conesa MA, Garcia Garcia T, et al Tumor-infiltrating immune cell profiles and their change after neoadjuvant chemotherapy predict response and prognosis of breast cancer. Breast Cancer Res. 2014;16(6):488 10.1186/s13058-014-0488-5 25432519 PMC4303200

[20] Melichar B, Studentova H, Kalabova H, Vitaskova D, Cermakova P, Hornychova H, et al Predictive and prognostic significance of tumor-infiltrating lymphocytes in patients with breast cancer treated with neoadjuvant systemic therapy. Anticancer Res. 2014;34(3):1115–25. . 24596349

[21] Pelekanou V, Barlow WE, Nahleh ZA, Wasserman B, Lo YC, von Wahlde MK, et al Tumor-Infiltrating Lymphocytes and PD-L1 Expression in Pre- and Posttreatment Breast Cancers in the SWOG S0800 Phase II Neoadjuvant Chemotherapy Trial. Molecular cancer therapeutics. 2018;17(6):1324–31. 10.1158/1535-7163.MCT-17-1005 . 29588392 PMC6548451

[22] Hamy AS, Bonsang-Kitzis H, De Croze D, Laas E, Darrigues L, Topciu L, et al Interaction between Molecular Subtypes and Stromal Immune Infiltration before and after Treatment in Breast Cancer Patients Treated with Neoadjuvant Chemotherapy. Clinical cancer research: an official journal of the American Association for Cancer Research. 2019;25(22):6731–41. 10.1158/1078-0432.CCR-18-3017 . 31515462

[23] Liu F, Li Y, Ren M, Zhang X, Guo X, Lang R, et al Peritumoral FOXP3(+) regulatory T cell is sensitive to chemotherapy while intratumoral FOXP3(+) regulatory T cell is prognostic predictor of breast cancer patients. Breast Cancer Res Treat. 2012;135(2):459–67. 10.1007/s10549-012-2132-3 . 22842982

[24] Ladoire S, Arnould L, Apetoh L, Coudert B, Martin F, Chauffert B, et al Pathologic complete response to neoadjuvant chemotherapy of breast carcinoma is associated with the disappearance of tumor-infiltrating foxp3+ regulatory T cells. Clin Cancer Res. 2008;14(8):2413–20. 10.1158/1078-0432.CCR-07-4491 . 18413832

[25] Ladoire S, Mignot G, Dabakuyo S, Arnould L, Apetoh L, Rebe C, et al In situ immune response after neoadjuvant chemotherapy for breast cancer predicts survival. J Pathol. 2011;224(3):389–400. 10.1002/path.2866 . 21437909

[26] Miyashita M, Sasano H, Tamaki K, Hirakawa H, Takahashi Y, Nakagawa S, et al Prognostic significance of tumor-infiltrating CD8+ and FOXP3+ lymphocytes in residual tumors and alterations in these parameters after neoadjuvant chemotherapy in triple-negative breast cancer: a retrospective multicenter study. Breast Cancer Res. 2015;17:124 10.1186/s13058-015-0632-x 26341640 PMC4560879

[27] Ruffell B, Au A, Rugo HS, Esserman LJ, Hwang ES, Coussens LM. Leukocyte composition of human breast cancer. Proc Natl Acad Sci U S A. 2012;109(8):2796–801. 10.1073/pnas.1104303108 21825174 PMC3287000

[28] Dieci MV, Radosevic-Robin N, Fineberg S, van den Eynden G, Ternes N, Penault-Llorca F, et al Update on tumor-infiltrating lymphocytes (TILs) in breast cancer, including recommendations to assess TILs in residual disease after neoadjuvant therapy and in carcinoma in situ: A report of the International Immuno-Oncology Biomarker Working Group on Breast Cancer. Semin Cancer Biol. 2017 10.1016/j.semcancer.2017.10.003 . 29024776

[29] Symmans WF, Peintinger F, Hatzis C, Rajan R, Kuerer H, Valero V, et al Measurement of residual breast cancer burden to predict survival after neoadjuvant chemotherapy. J Clin Oncol. 2007;25(28):4414–22. 10.1200/JCO.2007.10.6823 . 17785706

[30] Gnant M, Harbeck N, Thomssen C. St. Gallen 2011: Summary of the Consensus Discussion. Breast Care (Basel). 2011;6(2):136–41. 10.1159/000328054 21633630 PMC3100376

[31] Zitvogel L, Apetoh L, Ghiringhelli F, Andre F, Tesniere A, Kroemer G. The anticancer immune response: indispensable for therapeutic success? J Clin Invest. 2008;118(6):1991–2001. 10.1172/JCI35180 18523649 PMC2396905

[32] Mattarollo SR, Loi S, Duret H, Ma Y, Zitvogel L, Smyth MJ. Pivotal role of innate and adaptive immunity in anthracycline chemotherapy of established tumors. Cancer Res. 2011;71(14):4809–20. 10.1158/0008-5472.CAN-11-0753 . 21646474

[33] Carson WE 3rd, Shapiro CL, Crespin TR, Thornton LM, Andersen BL. Cellular immunity in breast cancer patients completing taxane treatment. Clinical cancer research: an official journal of the American Association for Cancer Research. 2004;10(10):3401–9. 10.1158/1078-0432.CCR-1016-03 . 15161695

