# Supplementary material for: Changes and prognostic values of tumor-infiltrating lymphocyte subsets after primary systemic therapy in breast cancer
Source: PLoS One. 2020 May 13;15(5):e0233037. doi: 10.1371/journal.pone.0233037 (PMC7219779; doi:10.1371/journal.pone.0233037)
Supplement: S2 Table — (DOCX) [file pone.0233037.s002.docx]

| **Clinicopathological**  **characteristics** | **Post-PST CD8+/CD4+ T cell ratio** | | ***p*-value** | **Post-PST FOXP3+/CD8+ T cell ratio** | | ***p*-value** | **Post-PST FOXP3+/CD4+ T cell ratio** | | ***p*-value** |
| --- | --- | --- | --- | --- | --- | --- | --- | --- | --- |
|  | **Low** | **High** |  | **Low** | **High** |  | **Low** | **High** |  |
|  | **No. (%)** | **No. (%)** |  | **No. (%)** | **No. (%)** |  | **No. (%)** | **No. (%)** |  |
| ypT stage |  |  | 0.808 |  |  | 0.228 |  |  | 0.013 |
| T1 | 40 (51.9) | 39 (50.0) |  | 36 (46.2) | 43 (55.8) |  | 32 (41.0) | 47 (61.0) |  |
| T2-T4 | 37 (48.1) | 39 (50.0) |  | 42 (53.8) | 34 (44.2) |  | 46 (59.0) | 30 (39.0) |  |
| ypN stage |  |  | 0.263 |  |  | 0.011 |  |  | 0.004 |
| N0 | 25 (32.5) | 19 (24.4) |  | 15 (19.2) | 29 (37.7) |  | 14 (17.9) | 30 (39.0) |  |
| N1-pN3 | 52 (67.5) | 59 (75.6) |  | 63 (80.8) | 48 (62.3) |  | 64 (82.1) | 47 (61.0) |  |
| RCB class |  |  | 0.468 |  |  | 0.127 |  |  | 0.002 |
| I-II | 35 (45.5) | 40 (51.3) |  | 33 (42.3) | 42 (54.5) |  | 28 (35.9) | 47 (61.0) |  |
| III | 42 (54.5) | 38 (48.7) |  | 45 (57.7) | 35 (45.5) |  | 50 (64.1) | 30 (39.0) |  |
| Estrogen receptor |  |  | 0.882 |  |  | 0.165 |  |  | 0.001 |
| Negative | 17 (22.1) | 18 (23.1) |  | 14 (17.9) | 21 (27.3) |  | 9 (11.5) | 26 (33.8) |  |
| Positive | 60 (77.9) | 60 (76.9) |  | 64 (82.1) | 56 (72.1) |  | 69 (88.5) | 51 (66.2) |  |
| Progesterone receptor |  |  | 0.564 |  |  | 0.472 |  |  | 0.091 |
| Negative | 32 (41.6) | 36 (46.2) |  | 32 (41.0) | 36 (46.8) |  | 29 (37.2) | 39 (50.6) |  |
| Positive | 45 (58.4) | 42 (53.8) |  | 46 (59.0) | 41 (53.2) |  | 49 (62.8) | 38 (49.4) |  |
| HER2 status |  |  | 0.250 |  |  | 0.772 |  |  | 0.404 |
| Negative | 64 (83.1) | 59 (75.6) |  | 61 (78.2) | 62 (80.5) |  | 64 (82.1) | 59 (76.6) |  |
| Positive | 13 (16.9) | 19 (24.4) |  | 17 (21.8) | 15 (19.5) |  | 14 (17.9) | 18 (23.4) |  |
| Ki-67 index |  |  | 0.684 |  |  | 0.011 |  |  | 0.067 |
| Low (<20%) | 54 (70.1) | 57 (73.1) |  | 63 (80.8) | 48 (62.3) |  | 61 (78.2) | 50 (64.9) |  |
| High (≥20%) | 23 (29.9) | 21 (26.9) |  | 15 (19.2) | 29 (37.7) |  | 17 (21.8) | 27 (35.1) |  |
| P53 overexpression |  |  | 0.765 |  |  | 0.450 |  |  | 0.012 |
| Absent | 55 (71.4) | 54 (69.2) |  | 57 (73.1) | 52 (67.5) |  | 62 (79.5) | 47 (61.0) |  |
| Present | 22 (28.6) | 24 (30.8) |  | 21 (26.9) | 25 (32.5) |  | 16 (20.5) | 30 (39.0) |  |

**S2 Table. Relationship between ratios of tumor-infiltrating lymphocyte subsets after primary systemic therapy and post-treatment clinicopathological characteristics of tumor**

*P* values were calculated by the chi-square or Fisher’s exact test.

PST, primary systemic therapy; HER2, human epidermal growth factor receptor 2
