# Supplementary material for: Changes and prognostic values of tumor-infiltrating lymphocyte subsets after primary systemic therapy in breast cancer
Source: PLoS One. 2020 May 13;15(5):e0233037. doi: 10.1371/journal.pone.0233037 (PMC7219779; doi:10.1371/journal.pone.0233037)
Supplement: S3 Table — (DOCX) [file pone.0233037.s003.docx]

**S3 Table. Survival analyses using TIL subset variables using total count**

| **Variable** | ***p*-value** |
| --- | --- |
| Pre-PST CD8+ TIL | 0.102 |
| Pre-PST CD4+ TIL | 0.912 |
| Pre-PST FOXP3+ TIL | 0.864 |
| Post-PST CD8+ TIL | 0.029 |
| Post-PST CD4+ TIL | 0.185 |
| Post-PST FOXP3+ TIL | 0.526 |
| pre-PST FOXP3+/CD8+ TIL ratio | 0.335 |
| pre-PST FOXP3+/CD4+ TIL ratio | 0.940 |
| pre-PST CD8+/CD4+ TIL ratio | 0.868 |
| post-PST FOXP3+/CD8+ TIL ratio | 0.168 |
| post-PST FOXP3+/CD4+ TIL ratio | 0.449 |
| post-PST CD8+/CD4+ TIL ratio | 0.072 |
| post-PST CD8+/pre-PST CD8+ TIL ratio | 0.352 |
| post-PST CD4+/pre-PST CD4+ TIL ratio | 0.320 |
| post-PST FOXP3+/pre-PST FOXP3 TIL ratio | 0.081 |

P values were calculated using log rank test.
